# Mitochondrial Profiling of Acute Myeloid Leukemia in the Assessment of Response to Apoptosis Modulating Drugs

**DOI:** 10.1371/journal.pone.0138377

**Published:** 2015-09-16

**Authors:** Jo Ishizawa, Kensuke Kojima, Teresa McQueen, Vivian Ruvolo, Dhruv Chachad, Graciela M. Nogueras-Gonzalez, Xuelin Huang, William E. Pierceall, E. J. Dettman, Michael H. Cardone, Sharon Shacham, Marina Konopleva, Michael Andreeff

**Affiliations:** 1 Section of Molecular Hematology and Therapy, Department of Leukemia, The University of Texas MD Anderson Cancer Center, Houston, Texas, United States of America; 2 Department of Biostatistics, Division of Quantitative Sciences, The University of Texas MD Anderson Cancer Center, Houston, Texas, United States of America; 3 Eutropics Pharmaceuticals, Cambridge, Massachusetts, United States of America; 4 Karyopharm Therapeutics, Boston, Massachusetts, United States of America; 5 Hematology, Respiratory Medicine and Oncology, Department of Medicine, Saga University, Saga, Japan; Roswell Park Cancer Institute, UNITED STATES

## Abstract

BH3 profiling measures the propensity of transformed cells to undergo intrinsic apoptosis and is determined by exposing cells to BH3-mimicking peptides. We hypothesized that basal levels of prosurvival BCL-2 family proteins may modulate the predictive power of BH3 profiling and termed it mitochondrial profiling. We investigated the correlation between cell sensitivity to apoptogenic agents and mitochondrial profiling, using a panel of acute myeloid leukemias induced to undergo apoptosis by exposure to cytarabine, the BH3 mimetic ABT-199, the MDM2 inhibitor Nutlin-3a, or the CRM1 inhibitor KPT-330. We found that the apoptogenic efficacies of ABT-199 and cytarabine correlated well with BH3 profiling reflecting BCL2, but not BCL-XL or MCL-1 dependence. Baseline BCL-2 protein expression analysis increased the ability of BH3 profiling to predict resistance mediated by MCL-1. By utilizing engineered cells with overexpression or knockdown of BCL-2 family proteins, Ara-C was found to be independent, while ABT-199 was dependent on BCL-XL. BCL-2 and BCL-XL overexpression mediated resistance to KPT-330 which was not reflected in the BH3 profiling assay, or in baseline BCL-2 protein levels. In conclusion, mitochondrial profiling, the combination of BH3 profiling and prosurvival BCL-2 family protein analysis, represents an improved approach to predict efficacy of diverse agents in AML and may have utility in the design of more effective drug combinations.

## Introduction

Treatment outcomes for acute myeloid leukemia (AML) are generally better than those achievable for many other malignancies. However, AML patients frequently develop resistant disease demonstrating the limits of conventional chemotherapeutic agents like cytarabine (Ara-C). Numerous molecularly targeted agents have entered clinical trials as therapeutic candidates for AML, and an *in vitro* prognostic testing strategy to predict the putative anti-leukemic efficacy of these compounds would be highly desirable, and could lead to better decisions regarding combinatorial strategies and disease management.

BH3-only members of the BCL-2 family proteins consist of pro-apoptotic “sensitizers” (e.g., BAD, HRK, and NOXA) and “activators” (e.g., BIM, tBID, and PUMA), which modulate the effect of pro-survival BCL-2 family members, such as BCL-2, BCL-XL, and MCL-1 [1]. Disruption of the delicate balance of proapoptotic and antiapoptotic BCL-2 family proteins results in BAX/BAK activation and apoptosis [1].

BH3 profiling is a functional assay [2–4], that determines BCL-2 family dependence and mitochondrial priming by exposing cells to peptides that mimic BH3 domain proteins [5]. Thus, BH3 profiling theoretically predicts cell sensitivity to apoptosis by targeting anti-apoptotic Bcl-2 family protein with particular BH3-mimetic peptides. Previous reports have established that BH3 profiling can predict outcomes (both treatment response and survival) of hematological malignancies including myeloma, acute lymphoblastic leukemia and AML treated with conventional chemotherapy [3, 4, 6]. Also, BH3 profiling was reported to correlate with the sensitivity of malignant myeloid cells to molecularly targeted agents such as ABT-737 [4], ABT-199 [7], vorinostat [8] and 5-azacytidine [9].

Alternatively, incorporating quantitation of basal expression levels of BCL-2 family proteins measured by classical methods (e.g., immunoblotting) may provide additional information for the assessment of sensitivity or resistance of cells to chemotherapeutic agents. While it remains to be fully understood how drug–induced cell death depends on each BCL-2 family member, basal BCL-2 protein expression levels are known to correlate with ABT-199 [7] or ABT-737 [10] sensitivity, and MCL-1 protein expression levels correlate with resistance [7, 10, 11] to these agents.

Therefore, we hypothesized that mitochondrial profiling, i.e. the combined assessment of BH3 profiling and of basal expression levels of various BCL-2 family proteins, is a promising tool for categorizing the dependence of chemotherapeutic agents on BCL-2 and/or BH3-only proteins for induction of apoptosis. We investigated the correlations among factors related to cell death and BCL-2 family proteins in AML by: 1) analysis of apoptosis of AML cells by four different anti-leukemia compounds: cytarabine (Ara-C), the BH3-mimetic ABT-199, the MDM2-inhibitor Nutlin-3a, and the XPO1-inhibitor KPT-330; 2) BH3 profiling of the AML cells; and 3) determination of basal protein expression levels of BCL-2, MCL-1, and BCL-XL (Figure A in S1 File).

p53 activation induces NOXA and PUMA which neutralize MCL-1 and BCL-XL. Therefore, it seems obvious, but has not yet been tested whether BH3 profiling could reveal particular characteristics of p53-mediated apoptosis. We selected Nutlin-3a and KPT-330 as agents that induce p53-mediated apoptosis as potential mechanism of action [12, 13]. It is not known if basal expression of p53, or p53 mutational status, affects BH3 profiling. In this study, we therefore also investigated the correlation between p53 function and BH3 profiling by denoting p53 mutational status and by generating p53-silenced AML cell lines.

Using BH3 profiling, we confirmed the BCL-2 dependence of apoptosis induced by ABT-199, and also unexpectedly found that Ara-C–induced apoptosis is BCL-2 dependent. More surprisingly, apoptosis induced by Nutlin-3a or KPT-330 did not show any specific correlation with BH3 profiling. Therefore, the characterization of apoptosis induced by the four study agents was insufficient using only BH3 profiling. Additional assessment of prosurvival BCL-2 family expression showed that MCL-1 protein expression levels predicted resistance to Ara-C and to ABT-199. Furthermore, we found that overexpressed or downregulated expression of BCL-2, BCL-XL and MCL-1 were differentially associated with resistance to Ara-C, ABT-199, Nutlin-3a or KPT-330. Our study demonstrates that mitochondrial profiling, a combined assessment of BH3 profiling and the basal expression of BCL-2 family proteins, is a potentially improved approach to predicting activity of antitumor agents.

## Materials and Methods

### Reagents

KPT-330 was synthesized at Karyopharm Therapeutics (Natick, MA). MDM2 antagonist Nutlin-3a was purchased from Cayman Chemical Company (Ann Arbor, MI), the BCL-2 inhibitor ABT-199 from Selleckchem (Houston, TX) and Ara-C was purchased from Sigma-Aldrich (St. Louis, MO).

### Cell culture

Cell lines were purchased from Leibniz-Institut DSMZ-Deutsche Sammlung von Mikroorganismen und Zellkulturen (Braunschweig, Germany) or the American Type Culture Collection (Manassas, VA) other than OCI-AML2, OCI-AML5, ML-2 and MUTZ-2 that were kindly provided by Dr. Sami Malek (University of Michigan, Ann Arbor, MI) and previously published [14]. Bcl-2 1863, and Mcl-1 1780 cells (Figure C in S1 File) is Bcl-2 or Mcl-1 overexpressing murine cells that were kindly provided by Dr. Anthony Letai (Dana Farber Cancer Institute, Boston, MA) and previously published [15]. The authenticity of the cell lines was confirmed by DNA fingerprinting with the short tandem repeat method, using a PowerPlex 16 HS System (Promega, Madison, WI) within 6 months prior to the experiments.

### Apoptosis analysis

Annexin V and propidium iodide (purchased from Sigma-Aldrich) binding assays were performed to determine apoptosis as described previously [12]. Apoptosis was quantified as the percent of annexin V-positive cells, and drug-specific apoptosis was calculated by the following formula: %specific apoptosis = (test−control) x 100 / (100−control).

### Immunoblot analysis

Immunoblot analysis of BCL-2 family proteins and p53 was conducted as described previously [12], and quantitated using the Odyssey imaging system (LI-COR Biotechnology, Lincoln, NE). The antibodies used are listed in supplemental materials and methods in the S2 File.

### BH3 profiling

BH3 profiling of AML cells was performed as described previously [5, 6]. Briefly, cells were permeabilized with digitonin (Sigma-Aldrich, St. Louis, MO), incubated with JC-1 (Enzo Life Sciences, Farmingdale, NY) and exposed to BH3 peptides (100 μmol/L BIM, 0.1 μmol/L BIM, 100 μmol/L PUMA, 10 μmol/L PUMA, 100 μmol/L NOXA, 100 μmol/L BAD, 100 μmol/L BMF, 100 μmol/L HRK, or 100 μmol/L PUMA2A) at room temperature. Dimethyl sulfoxide (DMSO, 1%) and carbonyl cyanide *m*-chlorophenyl hydrazone (CCCP; 10 μmol/L) were used as negative and positive controls, respectively (both purchased from Sigma-Aldrich). Fluorescence was measured using an Infinite plate reader (Tecan Group, Mannedorf, Switzerland) to detect JC-1 dye aggregation dispersal as a surrogate for depolarization. The area under the curve for each condition was measured and the proportion of depolarization was calculated using the following equation.

% Priming  = 1 − ([peptide  − CCCP] / [DMSO  − CCCP])

### Statistical analyses

Statistical analyses were performed using the SAS (version 9.2; SAS Institute Inc., Cary, NC), STATA/SE (version 11.2; Stata Corp. LP, College Station, TX) and Prism (version 6.0; GraphPad Software, La Jolla, CA) statistical software programs. For analysis of the correlation between BH3 profiling and the extent of apoptosis induced by each compound, mixed linear model methods with repeated measures were used. *P* values less than 0.05 were considered statistically significant. Unless otherwise indicated, values are expressed as the mean ± standard deviation (SD) of triplicate samples.

## Results

### BH3 profiling identifies Ara-C- and ABT-199-induced apoptosis as BCL-2 dependent, and Nutlin-3a- and KPT-330-induced apoptosis as BCL-2 independent

**Fig 1:**
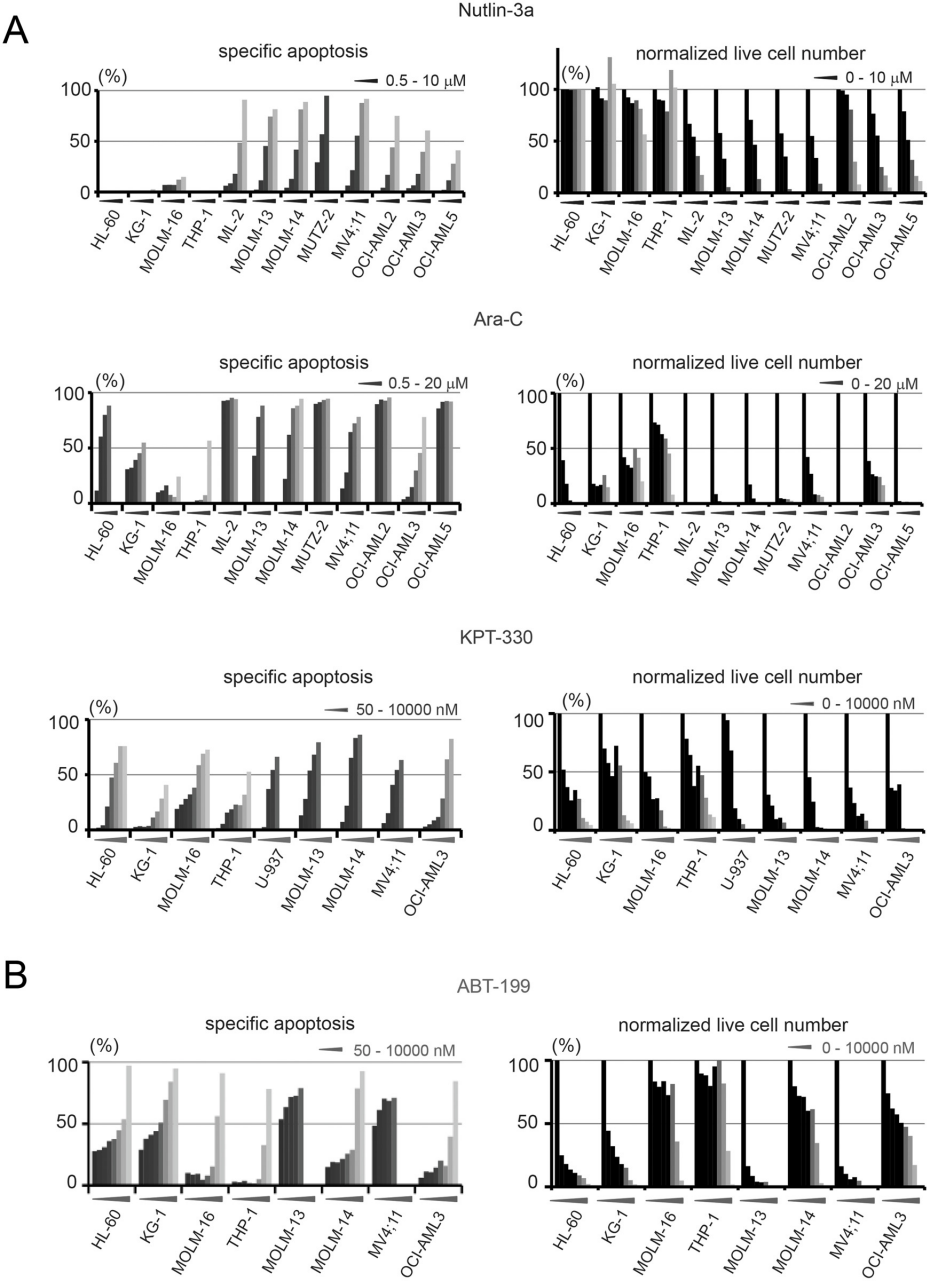
Apoptosis analysis of AML cell lines. The indicated cell lines were incubated with (A) Nutlin-3a (0, 0.5, 1.0, 2.5, 5, or 10 μM), Ara-C (0, 0.5, 1.0, 2.5, 5, 10, or 20 μM), KPT-330 (0, 50, 100, 250, 500, 1000 nM in U937, MOLM-13, MOLM-13 and MV4;11 cells. 0, 50, 100, 250, 500, 1000, 2500, 5000, and 10,000 nM in HL-60, KG-1, MOLM-16, THP-1, U-937 and OCI-AML3 cells), or (B) ABT-199 (0, 50, 100, 250, 500, 1000 nM in MOLM-13 and MV4;11 cells. 0, 50, 100, 250, 500, 1000, 2500, 5000, and 10,000 nM in HL-60, KG-1, MOLM-16, THP-1 and OCI-AML3 cells) for 48 hours. The annexin V-positive cell fractions and annexin V- and propidium iodide-negative live cells were counted using flow cytometry. The y axes of left graphs show the extent of drug-specific apoptosis, calculated as described in the method section. The standard deviations were confirmed to be less than 5% of each mean value by conducting three independent experiments in triplicate to assess the Annexin V-positive and live cell numbers.

We first assessed the sensitivity of the AML cell lines to Ara-C, ABT-199, Nutlin-3a, and KPT-330 by determining the fraction of annexin V positive cells and absolute numbers of Annexin V- and propidium iodide-negative live cells after 48 hour treatment (Fig 1). Cells were treated with previously reported and clinically relevant concentrations for all the four drugs [12, 16–18]. As reported previously [12, 13], Nutlin-3a and KPT-330 more effectively induced apoptosis in cells with wild-type p53 (OCI-AML3, MOLM-13 and MV4;11) (Fig 1A). In comparison, KPT-330 exerts its anti-proliferative effect even in cells with mutant p53, unlike Nutlin-3a (Fig 1A).

Because the anti-tumor effects of Ara-C, Nutlin-3a and KPT-330 encompass not only apoptogenic but also anti-proliferative effects in which cell cycle transit is inhibited independently of mitochondrial apoptosis, assessing the decrease in live cell numbers would result in overestimation of the cells’ sensitivity to these agents in terms of mitochondrial apoptosis, which is the main focus of the present study. For example, KG-1 cells were resistant to Ara-C–induced apoptosis (Fig 1A, left), but KG-1 cell growth was efficiently inhibited by Ara-C (Fig 1A, right). We observed similar results in OCI-AML3 cells treated with Nutlin-3a and KPT-330 and therefore postulated that the percentage of annexin V-positive cells is a better end point than the number of live cells in the assessment of apoptogenic effects of these three compounds (Fig 1A). Moreover, because ABT-199 induces apoptosis rapidly (within several hours), many of the annexin V positive cells have disintegrated after 48 hours. Therefore, assessing only annexin V positivity underestimates cell sensitivity to ABT-199. Assuming that ABT-199 theoretically does not have anti-proliferative effects unlike the other three compounds, we used viable cell counts (annexin V and propidium iodide double-negative cells) for drug-sensitivity indexing of apoptosis induced by ABT-199. For example, HL-60 and MOLM-14 appeared to have similar sensitivity to ABT-199 in terms of annexin V positivity (Fig 1B, left). However, HL-60 is one of the most ABT-199-sensitive cell lines, whereas MOLM-14 is one of the most ABT-199-resistant cell lines (Fig 1B, right).

**Fig 2:**
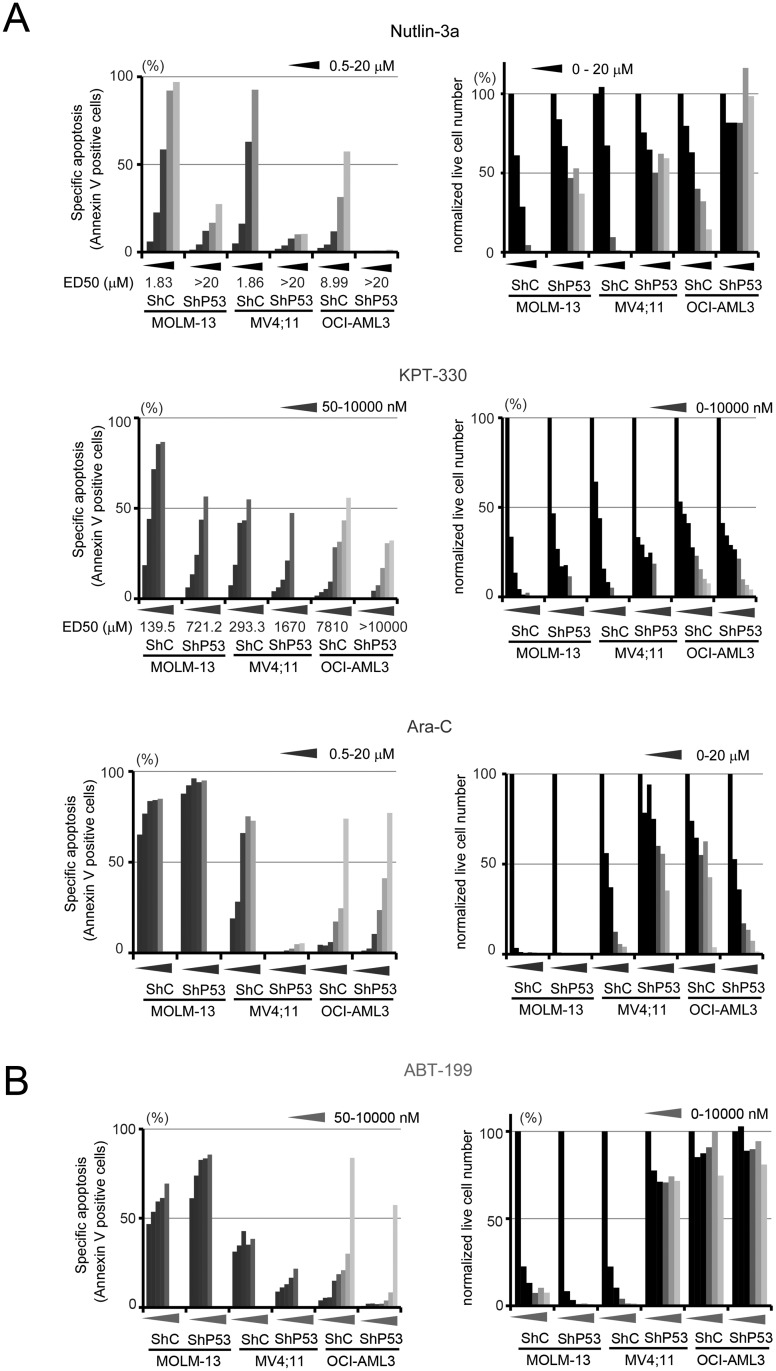
Apoptosis analysis of AML cells with p53 knockdown. MOLM-13, MV4;11 and OCI-AML3 cell lines transfected with a lentivirus carrying scrambled shRNA (ShC) or a shRNA specific for p53 (ShP53) were treated with (A) Nutlin-3a (0, 0.5, 1.0, 2.5, 5, or 10 μM), Ara-C (0, 0.5, 1.0, 2.5, 5, 10, or 20 μM), KPT-330 (0, 50, 100, 250, 500, 1000 nM in MOLM-13 and MV4;11 cells. 0, 50, 100, 250, 500, 1000, 2500, 5000, and 10,000 nM in OCI-AML3 lines), or (B) ABT-199 (0, 50, 100, 250, 500, 1000 nM in MOLM-13 and MV4;11 cells. 0, 50, 100, 250, 500, 1000, 2500, 5000, and 10,000 nM in OCI-AML3 lines) for 48 hours. The Annexin V-positive cell fractions and Annexin V- and propidium iodide-negative live cells were counted using flow cytometry. All experiments were performed in duplicate. The y axes of left graphs show the extent of drug-specific apoptosis, calculated as described in the method section.

We took advantage of 3 isogenic cell lines stably expressing p53-specific or control shRNA. p53 knockdown de-sensitized cells to p53 activators Nutlin-3a and KPT-330 (Fig 2), as previously reported [12, 13]. Surprisingly, p53 expression levels did not affect OCI-AML3 and MOLM-13 cell sensitivity to Ara-C or ABT-199, while p53 knockdown conferred resistance to Ara-C or ABT-199 in MV4;11 cells (Fig 2).

**Fig 3:**
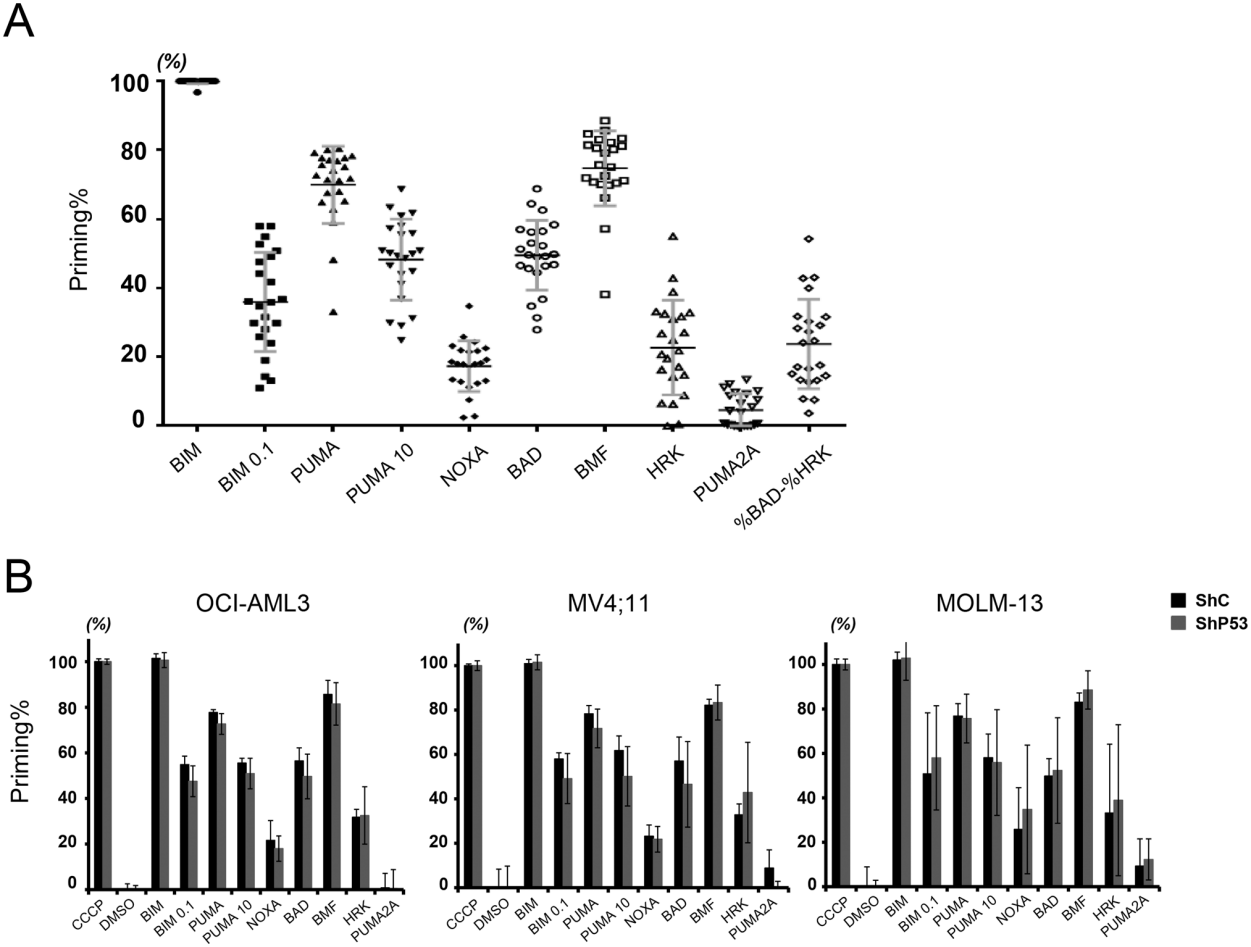
BH3 profiling of AML cell lines. (A) Distribution of mitochondrial priming to each BH3 peptide in 21 AML cell lines. Cells were exposed to 100 μmol/L BIM, 0.1 μmol/L BIM, 100 μmol/L PUMA, 10 μmol/L PUMA, 100 μmol/L NOXA, 100 μmol/L BAD, 100 μmol/L BMF, 100 μmol/L HRK, or 100 μmol/L PUMA2A at room temperature. Results are expressed as the mean ± standard error of the mean. (B) Comparison of BH3 profiling in p53-intact and -downregulated cell lines. Results are expressed as the mean ± SD. No significant differences in the priming values were observed in any pairs of the isogenic cell lines.

We then BH3 profiled 21 AML cell lines (Fig 3A and Table A in S1 File). The proportions of cells responding to each BH3 peptide (expressed as “%[BH3 peptide]”) differed widely enough to indicate their diversity in %[BIM 0.1 μM], %[PUMA 10 μM], %[NOXA], %[BAD], %[BMF] and %[HRK], compared with %[PUMA2A] as negative control (Fig 3A). Therefore, we utilized these values for further analysis. By using a mixed linear model, we analyzed the correlation between BH3 profiling and apoptosis induction described above (Table 1A). Although we expected %[BAD] to be correlated with the sensitivity of cells against ABT-199, we could not find a significant correlation in the cell lines examined. We speculated this could occur because the BAD peptide blocks not only BCL-2 but also BCL-XL. Thus, %[BAD] would not reflect BCL-2 dependence specifically.

**Table 1:** Correlation analysis between AML cell sensitivity to the four study agents and BH3 profiling using multivariate mixed linear models of both p53 wild-type and p53 mutant cell lines.

Correlation Analysis by Multivariate Mixed Linear Models								
	Nutlin-3a		Ara-C		KPT-330		ABT-199	
	β	p-value	β	p-value	β	p-value	β	p-value
**%BIM 0.1**	0.1	0.671	0.13	0.83	-0.04	0.921	-2.75	0.056
**% PUMA 10**	0.13	0.661	-0.34	0.642	0.78	0.088	0.38	0.734
**% NOXA**	0.34	0.555	0.95	0.506	1.04	0.124	2.39	0.249
**% BAD**	0.27	0.412	-0.26	0.756	0.08	0.875	-1.06	0.31
**% BMF**	0.34	0.199	-1	0.126	-0.54	0.453	1.98	0.271
**% HRK**	0.24	0.293	-1.05	0.056	0.49	0.22	0.76	0.366
**% BAD—% HRK**	-0.18	0.56	1.61	0.022	-0.88	0.109	-3.22	<0.001

Coefficient β for Nutlin-3a, KPT-330 and Ara-C is positive when the increase of %apoptosis is related to larger %[BH3 peptide], and β for ABT-199 is negative when the decrease of %live cell number is related to larger %[BH3 peptide].

To improve the parameter, we applied the algorithm, %[BAD]-%[HRK], which presumably would be better suited to predict BCL-2 dependence because preferential binding of HRK to BCL-XL has been reported [7, 19–22]. As expected, ABT-199 efficacy correlated positively with %[BAD]—%[HRK] (β = -3.22, p < 0.001, which means that, if the value of %[BAD]—%[HRK] increases by 1%, live cell number after treatment with ABT-199 decreases by 3.22%), which is compatible with BCL-2 dependence of ABT-199–induced apoptosis. Ara-C sensitivity unexpectedly also showed a similar correlation with %[BAD]—%[HRK] (β = 1.61, p < 0.05, which means that, if the value of %[BAD]—%[HRK] increases by 1%, apoptosis after treatment with Ara-C increases by 1.61%), indicating that this agent is also BCL-2 dependent, albeit with diminished specificity relative to ABT-199.

The KPT-330 sensitivity in p53 wild-type cell lines showed a borderline positive correlation with %[PUMA 10 μM] (β = 0.92, p = 0.054), while the correlation of KPT-330 sensitivity with %[PUMA 10mM] in all of the cell lines, including p53 mutant lines, was not significant (β = 0.78, p = 0.088) (Table 2). This may be explained by the notion that KPT-330 induces PUMA through p53 activation, however unexpectedly, Nutlin-3a–induced apoptosis, even though it is more specifically p53-mediated than that induced by KPT-330, did not correlate with any of the BH3 peptides, not even with p53 targets PUMA and NOXA.

**Table 2:** Correlation analysis between AML cell sensitivity to the four study agents and BH3 profiling using multivariate mixed linear models using only p53 wild-type cell lines treated with Nutlin-3a or KPT-330, compared with the analysis of all cell lines.

Correlation Analysis by Multivariate Mixed Linear Models								
	Nutlin-3a				KPT-330			
	all cell lines		only p53 wt lines		all cell lines		only p53 wt lines	
	β	p-value	β	p-value	β	p-value	β	p-value
**%BIM 0.1**	0.1	0.671	0.12	0.75	-0.04	0.921	-0.35	0.314
**% PUMA 10**	0.13	0.661	-0.12	0.801	0.78	0.088	**0.92**	**0.054**
**% NOXA**	0.34	0.555	-0.18	0.897	1.04	0.124	0.69	0.347
**% BAD**	0.27	0.412	0.24	0.712	0.08	0.875	0.03	0.961
**% BMF**	0.34	0.199	0.35	0.384	-0.54	0.453	-0.91	0.21
**% HRK**	0.24	0.293	0.13	0.78	0.49	0.22	0.04	0.931
**% BAD—% HRK**	-0.18	0.56	0	0.994	-0.88	0.109	-0.04	0.946

Coefficient β for Nutlin-3a, KPT-330 and Ara-C is positive when the increase of %apoptosis is related to larger %[BH3 peptide], and β for ABT-199 is negative when the decrease of %live cell number is related to larger %[BH3 peptide].

An additional consideration was whether p53 levels would influence BH3 profiling. We compared the results of BH3 profiling between three sets of isogenic AML cell lines transduced with lentiviruses encoding p53-specific or scrambled shRNA. Surprisingly, there were no significant differences between the two groups in general, suggesting p53 independence in the regulation of mitochondrial priming (Fig 3B).

### BH3 profiling predicts BCL-2 dependence, but MCL-1 protein expression better correlates with MCL-1 dependence in AML than BH3 profiling

**Fig 4:**
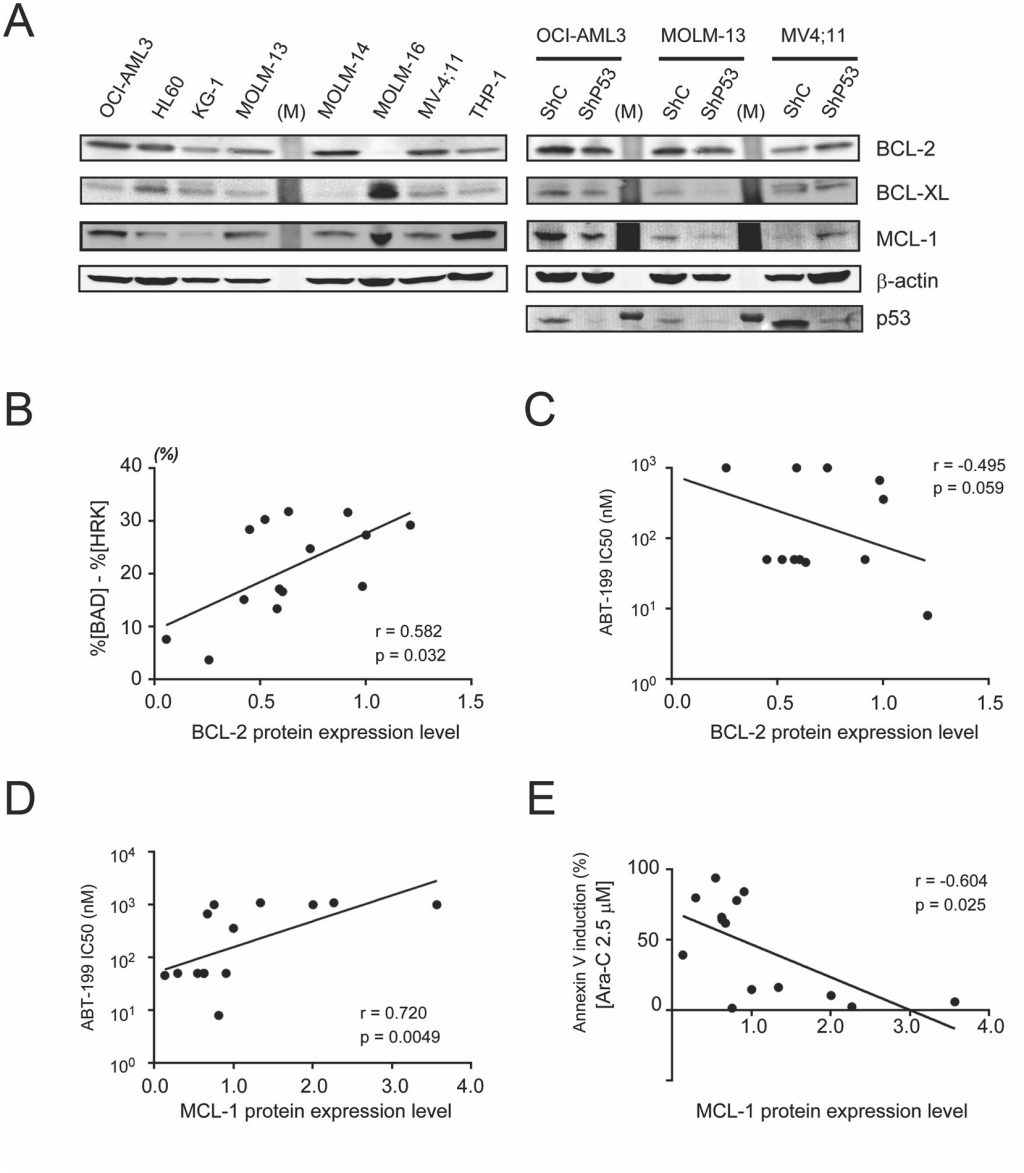
BCL-2, BCL-XL and MCL-1 protein expression, and its correlation with BH3 profiling or cell sensitivity to the study agents in AML cells. (A) Immunoblotting of BCL-2, BCL-XL and MCL-1 expression in AML cell lines. The lanes for protein ladder markers were represented as (M). (B) Correlation between BCL-2 protein expression and %[BAD]-%[HRK]. (C) Correlation between BCL-2 protein expression and the half-maximal inhibitory concentration (IC_50_) of ABT-199. (D) Correlation between MCL-1 protein expression and IC_50_ of ABT-199 in 14 AML cell lines treated with ABT-199 in Figs 1B and 2B. (E) Correlation between MCL-1 protein expression and apoptosis induced by Ara-C (2.5 μM). The values on the x-axes indicate protein expression normalized to the expression of each protein in OCI-AML3 cells. (Protein expression of BCL2 proteins normalized according to expression of beta-actin in each cell line before normalization to OCI-AML3 cells.)

Next, we assessed the correlation between protein expression of the prosurvival BCL-2 family proteins (BCL-2, BCL-XL or MCL-1) and sensitivity to BH3 peptides and to the four study drugs in representative 14 AML cell lines (described in Fig 4A). We determined BCL-2 family protein expression by immunoblotting (Fig 4A), and found that higher BCL-2 protein expression correlated well with higher value of our algorithm %[BAD]—%[HRK] (Fig 4B). Conversely, the correlation between BCL-2 protein expression and ABT-199 sensitivity was not statistically significant (Fig 4C). These results indicate that the algorithm, %[BAD]—%[HRK] was more strongly correlated with ABT-199 sensitivity than were BCL-2 protein expression levels. On the other hand, we observed no correlation between MCL-1 and %[NOXA] or between BCL-XL and %[HRK] despite their direct biological interactions, while increased expression of MCL-1 was associated with diminished cytotoxicity of ABT-199 (r = 0.720; p = 0.005, Fig 4D) and Ara-C (r = 0.604; p = 0.025, Fig 4E). These results demonstrate that the basal expression of MCL-1 protein was more strongly correlated with MCL-1-related resistance of these agents, as compared to BH3 profiling, whereas NOXA, the specific binding partner of MCL-1, did not predict resistance (Table 1). Of note, the correlations observed for BCL-XL protein levels were not statistically significant for any agent, and there were also no correlations between the sensitivities to Nutlin-3a and KPT-330 and expression of any prosurvival BCL-2 protein (Figure B in S1 File).

### Prosurvival BCL-2 protein family differentially regulates resistance of AML cells to chemotherapeutic agents

**Fig 5:**
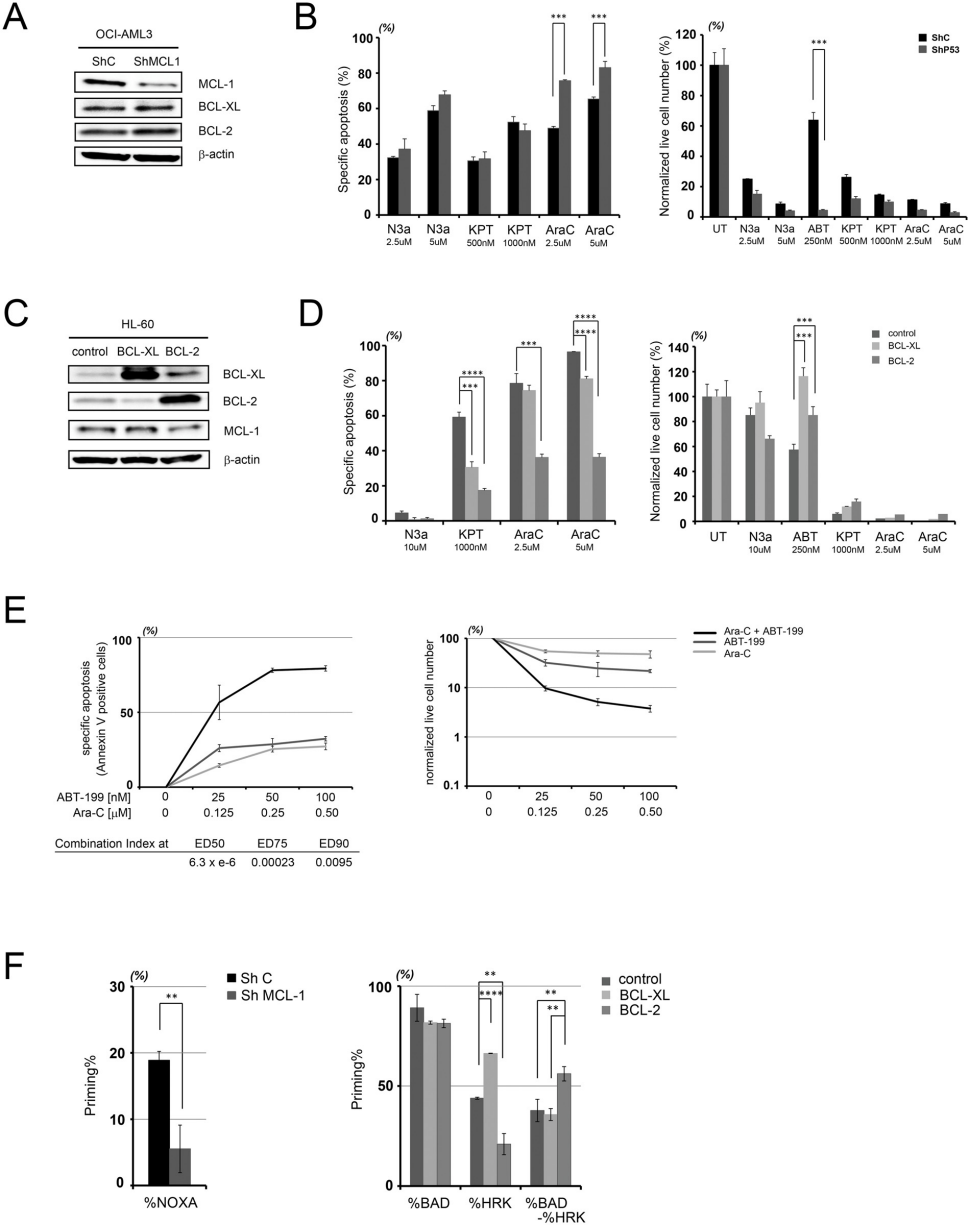
Effect of proapoptotic BCL-2 family protein expression on the sensitivity to Ara-C, Nutlin-3a (N3a), KPT-330 (KPT) and ABT-199 (ABT). (A) Immunoblot of BCL-2 family proteins in OCI-AML3 cells with control shRNA (ShC) or MCL-1 specific shRNA (ShMCL1). (B) Specific apoptosis induced by Nutlin-3a (2.5 or 5.0 μM), KPT-330 (500 or 1000 nM), Ara-C (2.5 or 5.0 μM) or ABT-199 (250 nM) in MCL-1-downregulated OCI-AML3 cells. ***P < 0.001 (C) Immunoblot of BCL-2 anti-apoptotic family proteins in HL-60 cells with overexpression of BCL-XL or BCL-2. (D) Specific apoptosis induced by Nutlin-3a (10 μM), KPT-330 (1000 nM), Ara-C (2.5 or 5.0 μM) or ABT-199 (250 nM) in BCL-XL- or BCL-2- overexpressing HL-60 cells. (E) Specific apoptosis induced by the combination of ABT-199 and Ara-C in KG-1 cells at 48 hours (left). Combination index was calculated by using CalcuSyn software (lower left). Annexin V- and PI-negative cells were counted as live cell number (right). (F) BH3 profiling of OCI-AML3 cells with control shRNA (ShC) or MCL-1 specific shRNA (ShMCL1), and HL-60 cells with control vector, overexpression of BCL-XL or BCL-2. The results are expressed as the mean ± SD. *P < 0.05, ** *P* < 0.01, ***P < 0.001, ****P < 0.0001.

To investigate how BCL-2, BCL-XL, and MCL-1 affect resistance of AML cells to the selected agents, we examined isogenic cell lines with altered expression of the BCL-2, BCL-XL, and MCL-1 genes. Because parental OCI-AML3 cells have relatively high MCL-1 protein expression levels, we generated isogenic OCI-AML3 cells where MCL-1 was downregulated by lentiviral infection using *MCL1*-specific shRNA (Fig 5A). As reported previously [7], MCL-1 downregulation sensitized OCI-AML3 cells to ABT-199 (Fig 5B, right), indicating that the degree of MCL-1 downregulation in our cell line was sufficient to assess drug resistance related to MCL-1. In addition, these cells were also more susceptible to Ara-C than were cells with unaltered MCL-1 (Fig 5B, left), which was consistent with the negative correlation between cell sensitivities to Ara-C and MCL-1 protein expression levels (Fig 3E). Conversely, MCL-1 downregulation did not affect apoptosis induced by Nutlin-3a or KPT-330, suggesting MCL-1 independence of these agents.

To investigate the effects of BCL-XL and BCL-2 overexpression on apoptosis, we utilized HL-60 cells that were engineered to overexpress each gene (Fig 5C). While Ara-C demonstrated similar sensitivity to ABT-199 related to MCL-1 knockdown, BCL-XL or BCL-2 overexpression had differential effects on the sensitivity to these agents: BCL-XL overexpression did not affect Ara-C–induced apoptosis to the extent that BCL-2 overexpression did. In fact, BCL-XL overexpression most effectively inhibited ABT-199–induced apoptosis. Together, these results indicate that BCL-2 overexpression can enhance resistance to Ara-C and KPT-330, while BCL-XL overexpression confers resistance to KPT-330 and ABT-199 (Fig 5D). This finding could provide rationale for combination therapies that would overcome BCL-2- and BCL-XL-mediated resistance. We then utilized the information generated by the assessment above to design mitochondrial profiling-based drug combinations, and combined ABT-199 with Ara-C in KG-1 cells, which express relatively high BCL-XL and are not highly sensitive to either ABT-199 nor Ara-C (Figs 1 and 4A). As expected, the combination was highly synergistic as it was designed to overcome the BCL-XL-dependent resistance to ABT-199 (Fig 5E).

We also conducted BH3 profiling using these engineered cells. As expected, BH3 profiling of MCL-1 knock-down OCI-AML3 cells, BCL-XL overexpressing and BCL-2 overexpressing HL-60 cells showed lower %[NOXA], higher %[HRK], and higher %[BAD]-%[HRK] respectively, compared to that of each corresponding control cell line (Fig 5F). This finding strongly supports the fundamental concept of this study, specifically that BH3 profiling predicts the BCL-2/MCL-1/BCL-XL dependence of the target cells.

## Discussion

Our findings comparing AML cell sensitivities to four apoptogenic compounds with BH3 profiling and prosurvival BCL-2 family protein expression suggest that BH3 profiling is a valuable tool to predict apoptosis induction by selected chemotherapy agents and targeted therapies. However, the additional assessment of prosurvival BCL-2 family proteins is required to detect resistance related to MCL-1 or BCL-XL. Thus, we conclude that mitochondrial profiling, by incorporating classical quantification of basal expression of BCL-2 family proteins with BH3 profiling, can characterize the dependence on BCL-2 family proteins of agent-specific apoptosis more precisely than could be done by BH3 profiling alone.

Results indicate that apoptosis induced by ABT-199, Ara-C, KPT-330, and Nutlin-3a exhibits various patterns of dependence on BCL-2 family proteins. As shown in previous work [7], ABT-199 induces BCL-2 dependent apoptosis in AML cells, and in the present study, ABT-199–induced apoptosis correlated well with the specific algorithm %[BAD]—%[HRK], and better than with %[BAD] as previously described [7]. On the other hand, a surprising result was the finding that Ara-C–induced apoptosis showed a similar correlation with BH3 profiling (%[BAD]—%[HRK]) and with expression levels of MCL-1 protein. This finding indicated that BH3 profiling has the potential to classify agents into unexpected categories, regardless of their known mechanisms of action. It was intriguing that p53 knockdown conferred resistance to Ara-C and ABT-199 in MV4;11 (Fig 2), perhaps explained by the observed up-regulation of MCL-1 in MV4;11 cells with p53 knockdown. Indeed, it has been shown that, in some circumstances, p53 represses MCL-1 in leukemia cells [23].

Importantly, for both Ara-C and ABT-199, there was a marked difference in the resistance conferred by BCL-XL overexpression, which was not detected by utilizing BH3 profiling alone. Specifically, ABT-199–induced apoptosis was inhibited more effectively by BCL-XL than by BCL-2, whereas inhibition of Ara-C–induced apoptosis by BCL-XL was minimal, while BCL-2 inhibited apoptosis much more effectively. Close inspection of published data revealed that BH3 profiling predicts cytochrome c release only in those samples who are very sensitive to ABT-199 in the low nanomolar range, and further demonstrate extreme variability at doses of ≥ 10 nM (Fig 5C, 5D and 5E) [7]. This suggests that BH3 profiling may not be able to predict the drug efficacy correctly because effects of BCL-XL or MCL-1 are not fully assessed in this assay. Specifically, BH3 profiling of NOXA does not predict BCL-XL resistance to ABT-199. Likewise, HRK profiling does not predict MCL-1 resistance of ABT-199. The broader assessment of mitochondrial profiling proposed in the present study may therefore enhance BH3 profiling.

Nutlin-3a showed the least correlation with all factors investigated in this study (e.g. BH3 profiling, basal protein expression of BCL-2 family proteins). Because wild-type p53, which almost exclusively mediates Nutlin-3a–induced apoptosis, is known to induce NOXA and PUMA, and perhaps also interacts directly with BCL-2 in its mitochondrial localization [1], we expected that p53-mediated cell death would correlate with BH3 profiling, and in particular with %priming in exposure to PUMA or NOXA-mimicking peptides. However, no correlation was observed with both BH3 profiling and prosurvival BCL-2 protein expression. These findings indicate that p53 induces apoptosis by differentially affecting a broad spectrum of BCL-2 proteins and that p53-mediated apoptosis possibly overcomes resistance to any single BH3-only protein. Another novel finding is that MCL-1 downregulation did not sensitize AML cells to Nutlin-3a or KPT-330. Conversely, the overexpression of BCL-XL or BCL-2 efficiently blocked KPT-330–induced cell death. Thus, the assays using cell lines with altered expression of BCL-2 family proteins provide additional useful information for the assessment of characteristics of apoptosis dependence of each agent.

Another surprising and novel finding was that p53 silencing in the AML cell lines with p53 wild type did not result in significant changes in BH3 profiling. This indicates that basal expression of p53 does not significantly impact mitochondrial priming, in contrast to the diverse effects on BCL-2 family proteins when p53 is induced under stress. This notion is supported by results from a currently ongoing Phase 1 study of ABT-199 in refractory chronic lymphoid leukemia patients [24], showing that similar objective response rates (ORR) (78%) were achieved in patients harboring chromosome 17p deletions (thus p53 deletion) with or without p53 mutations of the remaining allele, as compared to the ORR (79%) for the entire group. The clinical data indicate that BCL-2 dependence of ABT-199-induced apoptosis is not affected by p53 deletion, a finding that resonates well with results presented here.

On the other hand, it was not surprising that none of these 4 compounds showed correlations with either %[NOXA] or %[HRK]. As seen with the correlation between ABT-199 and %[BAD]-%[HRK] regarding BCL-2 dependence, other compounds that selectively inhibit MCL-1 or BCL-XL would be necessary to assess the usefulness of BH3 profiling with NOXA or HRK peptide exposure in assessing the MCL-1 or BCL-XL dependence of apoptosis, respectively. However, as shown in Fig 5F, BH3 profiling accurately detected the dependence on MCL-1, BCL-XL and BCL-2 in our MCL-1 downregulated and BCL-XL or BCL-XL overexpressed cell lines, further validating the approach to apoptosis prediction presented here.

The present study indicates that we can categorize selected existing and novel agents regarding their BCL2 dependence of apoptosis induction. Because counting annexin V-positive cells is only a snapshot of dead cells and does not reflect the dynamics of the apoptosis process, we utilized enumeration of live cells to assess cell sensitivity to apoptogenic agents, in particular ABT-199. Therefore, in order to apply mitochondrial profiling to future novel agents, one should determine the optimal and biologically relevant methods for assessing apoptosis. In addition, application of this method to primary AML samples may require modifications. In the present study, we used a plate reader to measure %priming, which assesses the bulk population of cells, which is acceptable because cell lines are considered to be much more homogeneous than primary cells. However, giving that heterogeneity is a hallmark of AMLs, BH3 profiling by flow cytometry would be superior for primary AML cells. We have reported that, by the flow cytometry method, BH3 profiling for a specific subpopulation (CD45+CD20-CD3- cells) can be performed. But we are also establishing here that the plate-reader-method provides results very similar to those obtained by flow cytometry (Figure C in S1 File).

Once we systemically develop a database containing proposed components of intrinsic apoptosis (e.g. BH3 profiling, cell sensitivity to each agent and BCL-2 family protein expression in targeted cells), one could generate a rational design of combination therapies by selecting differently categorized agents. For example, considering that Ara-C–induced apoptosis was not significantly affected by BCL-XL overexpression compared to ABT-199–induced apoptosis, we have provided rationale for its combination with ABT-199, which shows dramatic synergistic effects. This concept is presently being tested in a clinical trial combining ABT-199 with Ara-C. As we demonstrated that p53-mediated apoptosis inducers (e.g. Nutlin-3a and KPT-330) do not rely on just a limited set of BH3-only proteins, these agents have potential to be combined with other agents that exert an apoptogenic effect with dependence on a specific BCL-2 family protein (e.g. Ara-C and ABT-199). Indeed, we reported that the combination of ABT-737 and Nutlin [25] and of ABT-199 and the MDM2 inhibitor RG-7388 [26] synergistically induce apoptosis in AML cell lines and primary patient cells and overcome inherent or acquired resistance to ABT-199.

In conclusion, the novel technology employed in BH3-profiling, in conjunction with determination of the baseline expression levels of BCL-2 family members, provides useful insights into the mode of action of novel anti-cancer agents in inducing apoptosis and should be tested prospectively in Phase 1 clinical trials of novel agents targeting apoptosis pathways.

## Supporting Information

S1 File Supplemental Table and Figures. **Table A**. **BH3 profiling of 21 AML cell lines.** The numbers indicate the mean of %depolarization (1−[(Each peptide−CCCP) / (DMSO−CCCP)]) in triplicates per each condition. **Figure A**. **A schema of mitochondrial profiling. Figure B**. **BCL-2, BCL-XL, and MCL-1 protein expression and its correlation with AML cell sensitivity to the study agents.** (A) Correlation of BCL-XL and BCL-2 protein expression with cell sensitivity to AraC. (B and C) Correlation of BCL-2, BCL-XL and MCL-1 protein expression with cell sensitivity to Nutlin-3a (B) and KPT-330 (C). (D) Correlation of BCL-XL protein expression with cell sensitivity to ABT-199. The values on x-axes indicate protein expression normalized to protein expression level of each protein in OCI-AML3 cells. (Protein expression of BCL2 proteins normalized to expression of beta-actin in each cell line before normalization to OCI-AML3 cells.) **Figure C. A correlation of %priming between a flow cytometry based method and a plate reader method.** MOLM13, Bcl-2 1863, and Mcl-1 1780 cells were examined using BH3 profiling with a flow cytometry (FC) based method (overall MFI of the JC-1 red stain) and a plate reader method (Overall JC-1 red fluorescence throughout the entire plate read). The levels of priming for each of the peptides were compared across these three cell lines. The cell types are indicated by the symbol shape in the above graph, and the peptides are represented by the color of the symbol. Overall, the Pearson correlation, R squared, value was 0.917 (p = 1.6e-9). This correlation appears to be general across each peptide among these three different cell lines. The linear regression model fit is illustrated in the blue line with the 95% confidence intervals of that fit shown by the shaded region.
(DOCX)

S2 File Supplemental Materials and Methods. (DOCX)
