# Supplementary material for: Mitochondrial Profiling of Acute Myeloid Leukemia in the Assessment of Response to Apoptosis Modulating Drugs
Source: PLoS One. 2015 Sep 16;10(9):e0138377. doi: 10.1371/journal.pone.0138377 (PMC4573975; doi:10.1371/journal.pone.0138377)
Supplement: S1 File — Table A. BH3 profiling of 21 AML cell lines. The numbers indicate the mean of %depolarization (1−[(Each peptide−CCCP) / (DMSO−CCCP)]) in triplicates per each condition. Figure A. A schema of mitochondrial profiling. Figure B. BCL-2, BCL-XL, and MCL-1 protein expression and its correlation with AML cell sensitivity to the study agents. (A) Correlation of BCL-XL and BCL-2 protein expression with cell sensitivity to AraC. (B and C) Correlation of BCL-2, BCL-XL and MCL-1 protein expression with cell sensitivity to Nutlin-3a (B) and KPT-330 (C). (D) Correlation of BCL-XL protein expression with cell sensitivity to ABT-199. The values on x-axes indicate protein expression normalized to protein expression level of each protein in OCI-AML3 cells. (Protein expression of BCL2 proteins normalized to expression of beta-actin in each cell line before normalization to OCI-AML3 cells.) Figure C. A correlation of %priming between a flow cytometry based method and a plate reader method. MOLM13, Bcl-2 1863, and Mcl-1 1780 cells were examined using BH3 profiling with a flow cytometry (FC) based method (overall MFI of the JC-1 red stain) and a plate reader method (Overall JC-1 red fluorescence throughout the entire plate read). The levels of priming for each of the peptides were compared across these three cell lines. The cell types are indicated by the symbol shape in the above graph, and the peptides are represented by the color of the symbol. Overall, the Pearson correlation, R squared, value was 0.917 (p = 1.6e-9). This correlation appears to be general across each peptide among these three different cell lines. The linear regression model fit is illustrated in the blue line with the 95% confidence intervals of that fit shown by the shaded region. (DOCX) [file pone.0138377.s001.docx]

# **S1 File: Supplemental Table and Figures**

**Table A. BH3 profiling of 21 AML cell lines.**

| **Cell Line** | **%BIM** | **%BIM 0.1** | **%PUMA** | **%PUMA 10** | **%NOXA** | **%BAD** | **%BMF** | **%HRK** | **%PUMA2A** | **%BAD-%HRK** |
| --- | --- | --- | --- | --- | --- | --- | --- | --- | --- | --- |
| **OCI-AML3 Parental** | 100.00 | 29.85 | 80.09 | 49.37 | 21.96 | 49.23 | 84.69 | 21.88 | 11.25 | 27.35 |
| **OCI-AML3 ShC** | 100.00 | 54.88 | 77.70 | 55.48 | 21.56 | 56.47 | 85.64 | 31.73 | 0.72 | 24.74 |
| **OCI-AML3 ShP53** | 100.00 | 47.52 | 72.66 | 50.91 | 17.96 | 49.60 | 81.46 | 32.50 | 0.40 | 17.10 |
| **HL-60** | 100.00 | 35.89 | 64.88 | 41.20 | 7.60 | 44.51 | 75.69 | 14.23 | 0.00 | 30.28 |
| **KG-1** | 100.00 | 36.73 | 65.21 | 46.50 | 12.39 | 51.33 | 71.11 | 19.56 | 0.38 | 14.57 |
| **Molm 13 Parental** | 100.00 | 28.06 | 77.07 | 48.65 | 19.24 | 56.26 | 70.04 | 27.06 | 5.49 | 31.77 |
| **Molm 13 ShC** | 100.00 | 50.82 | 76.81 | 58.07 | 25.81 | 49.79 | 83.04 | 33.17 | 9.27 | 12.74 |
| **Molm 13 ShP53** | 100.00 | 57.96 | 75.68 | 55.86 | 34.75 | 52.27 | 88.51 | 38.87 | 12.23 | 29.20 |
| **Molm 16** | 100.00 | 29.88 | 71.40 | 50.84 | 22.56 | 62.59 | 75.07 | 55.00 | 13.47 | 16.62 |
| **MV4;11 Parental** | 100.00 | 19.10 | 71.14 | 44.85 | 12.84 | 46.41 | 70.73 | 14.79 | 9.79 | 13.40 |
| **MV4;11 ShC** | 100.00 | 24.06 | 75.15 | 44.03 | 18.28 | 49.13 | 72.53 | 20.76 | 7.60 | 7.59 |
| **MV4;11 ShP53** | 100.00 | 49.13 | 71.75 | 50.15 | 21.79 | 46.55 | 83.38 | 42.85 | 0.00 | 31.62 |
| **THP-1** | 99.94 | 25.82 | 67.94 | 24.90 | 11.28 | 31.39 | 81.11 | 16.27 | 0.82 | 24.18 |
| **Molm 14** | 96.77 | 11.05 | 33.09 | 68.62 | 13.12 | 34.77 | 66.16 | 17.16 | 0.00 | 28.37 |
| **OCI-AML2** | 100.00 | 14.35 | 59.03 | 36.86 | 18.06 | 46.81 | 57.18 | 0.49 | 10.22 | 3.69 |
| **OCI-AML5** | 99.71 | 13.16 | 48.18 | 31.20 | 17.42 | 27.94 | 38.14 | 0.00 | 4.01 | 15.12 |
| **ML-2** | 100.00 | 44.22 | 67.56 | 49.95 | 13.49 | 53.05 | 69.81 | 8.81 | 4.44 | 43.06 |
| **MUTZ-2** | 100.00 | 36.14 | 62.80 | 29.95 | 18.63 | 45.66 | 70.45 | 6.33 | 6.60 | 42.83 |
| **U-937** | 100.00 | 52.71 | 77.70 | 57.30 | 24.35 | 58.38 | 80.61 | 31.00 | 0.40 | 39.98 |
| **NB-4** | 100.00 | 34.68 | 79.25 | 60.93 | 2.48 | 64.47 | 80.19 | 26.73 | 0.00 | 17.62 |
| **SIG-M5** | 100.00 | 31.57 | 74.08 | 29.08 | 17.91 | 36.73 | 71.89 | 6.52 | 0.00 | 54.24 |

*The numbers indicate the mean of %depolarization (1 – [(Each peptide – CCCP) / (DMSO – CCCP)]) in triplicates per each condition.

**
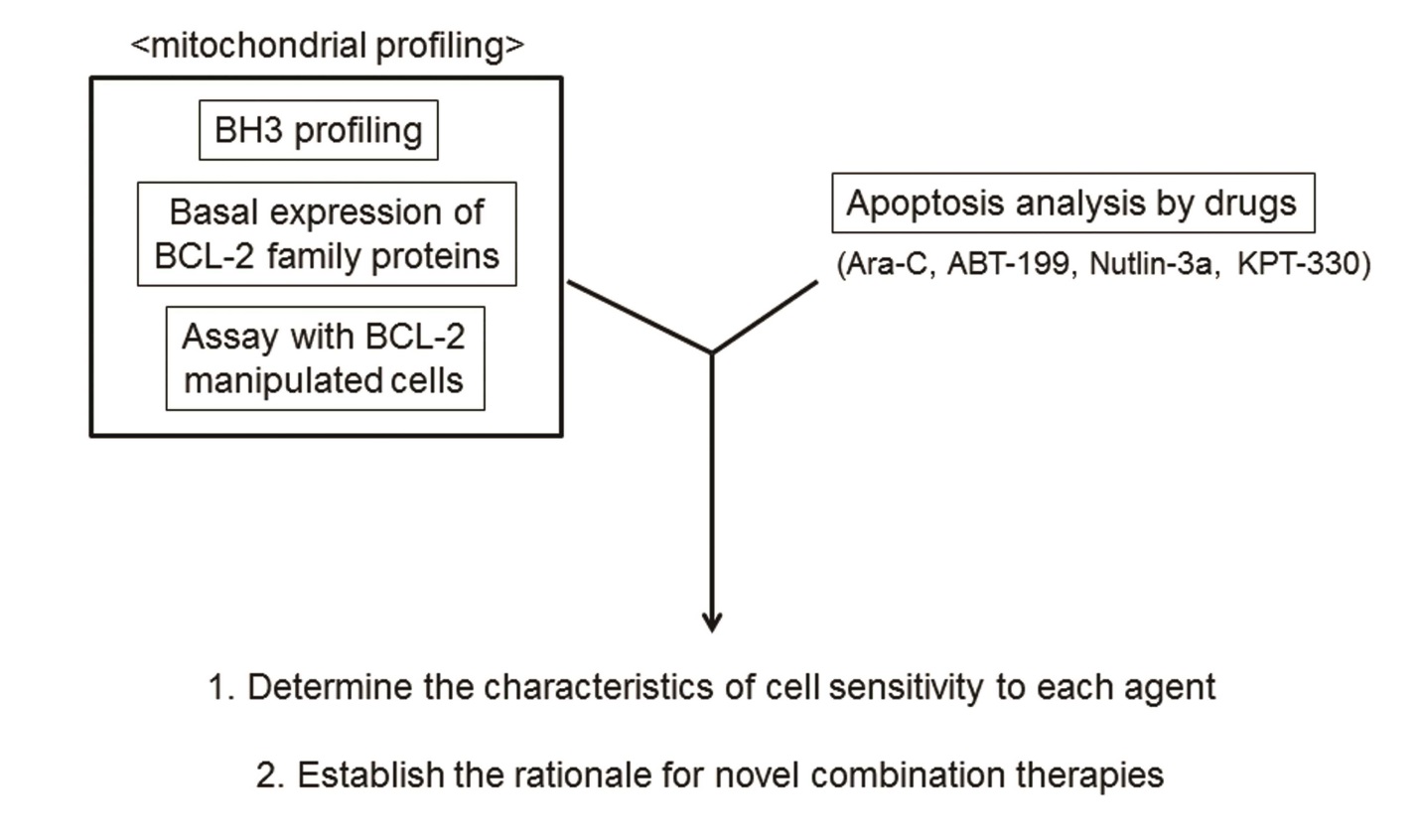
**

**Figure A. Schema of mitochondrial profiling.**

**
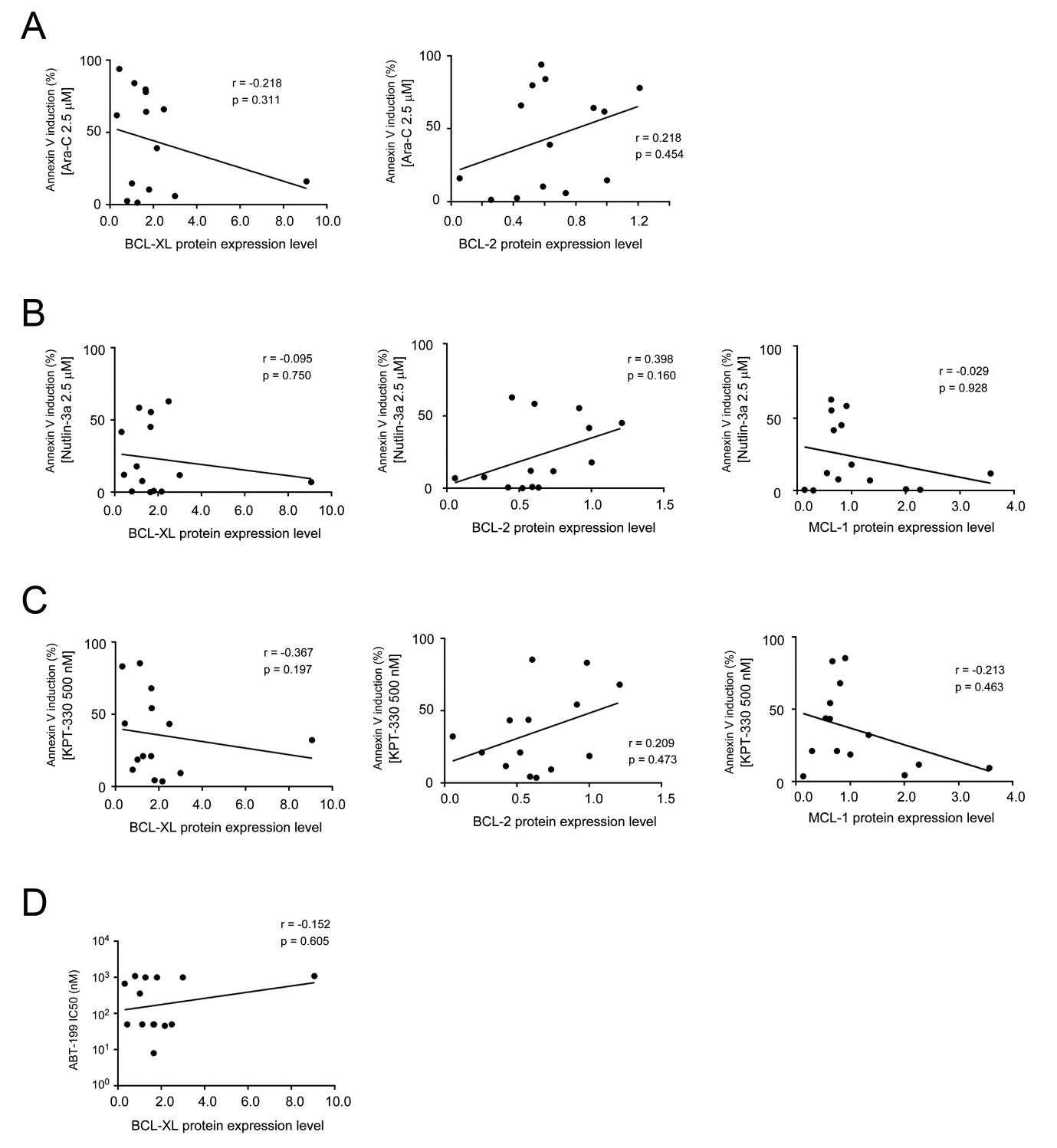
**

**Figure B.** **BCL-2, BCL-XL and MCL-1 protein expression and its correlation with AML cell sensitivity to the study agents.** (A) Correlation of BCL-XL and BCL-2 protein expression with cell sensitivity to AraC. (B and C) Correlation of BCL-2, BCL-XL and MCL-1 protein expression with cell sensitivity to Nutlin-3a (B) and KPT-330 (C). (D) Correlation of BCL-XL protein expression with cell sensitivity to ABT-199. The values on x-axes indicate protein expression normalized to protein expression level of each protein in OCI-AML3 cells. (Protein expression of BCL2 proteins normalized to expression of beta-actin in each cell line before normalization to OCI-AML3 cells.)

**
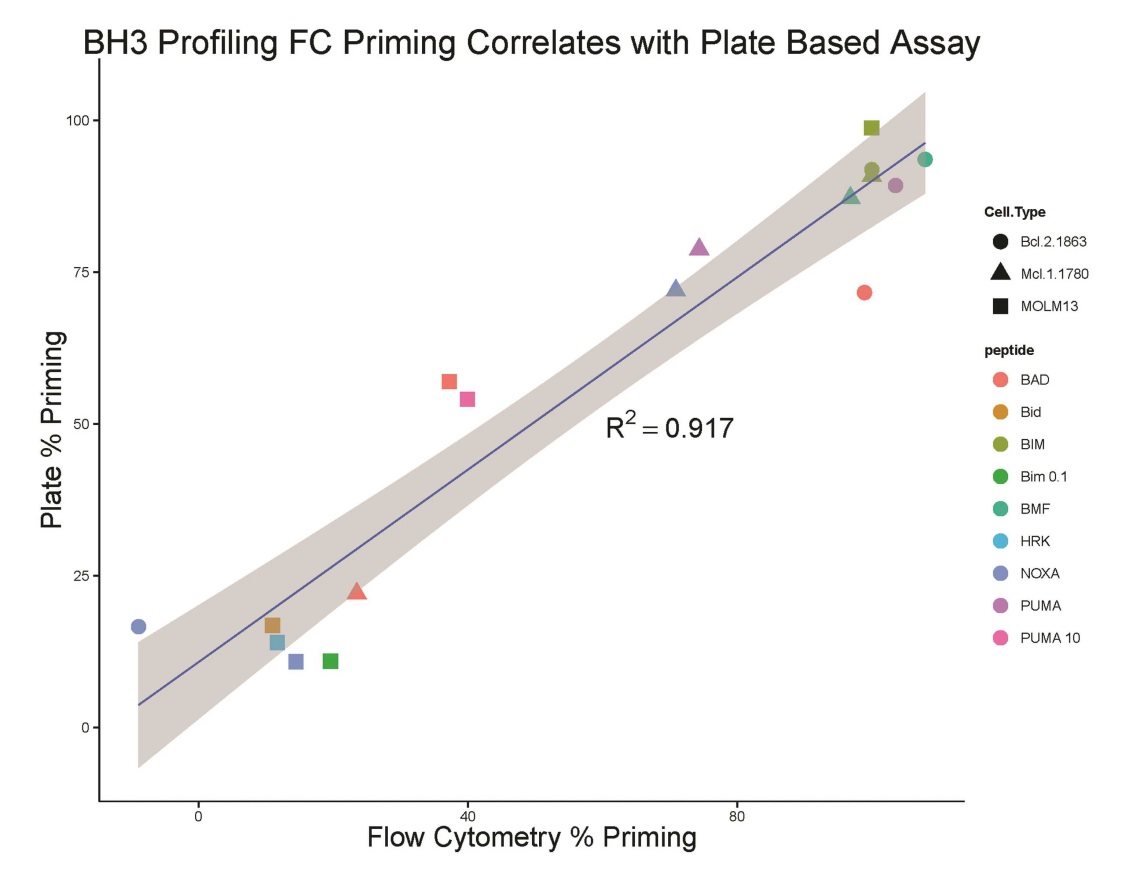
**

**Figure C.** **Correlation of %priming between a flow cytometry based method and a plate reader method.** MOLM13, Bcl-2 1863, and Mcl-1 1780 cells were examined using BH3 profiling with a flow cytometry (FC) based method (overall MFI of the JC-1 red stain) and a plate reader method (Overall JC-1 red fluorescence throughout the entire plate read). The levels of priming for each of the peptides were compared across these three cell lines. The cell types are indicated by the symbol shape in the above graph, and the peptides are represented by the color of the symbol. Overall, the Pearson correlation, R squared, value was 0.917 (p = 1.6e-9). This correlation appears to be general across each peptide among these three different cell lines. The linear regression model fit is illustrated in the blue line with the 95% confidence intervals of that fit shown by the shaded region.
