# Supplementary material for: Mitochondrial Profiling of Acute Myeloid Leukemia in the Assessment of Response to Apoptosis Modulating Drugs
Source: PLoS One. 2015 Sep 16;10(9):e0138377. doi: 10.1371/journal.pone.0138377 (PMC4573975; doi:10.1371/journal.pone.0138377)
Supplement: S2 File — (DOCX) [file pone.0138377.s002.docx]

**S2 File: Supplemental Materials and Methods**

**Cell culture and lentiviral infection of shRNAs.**

The cells were cultured in RPMI 1640 medium containing 10% fetal bovine serum. ML-2, MOLM-13, MOLM-14, MUTZ-2, MV4;11, OCI-AML2, OCI-AML3, OCI-AML5 were cell lines with wild-type p53, while HL-60, KG-1, MOLM-16, THP-1 and U-937 are p53 deficient, either p53 mutant or deleted. Cells were harvested at log-phase growth, plated at a density of 2.0 x 10^5^ per mL and treated with the indicated compounds. MOLM-13, MV4;11, and OCI-AML3 cells were transduced with lentivirus delivering p53-specific short hairpin RNA (shRNA; nucleotides 611-629, RefSeq NM000546) and stable shRNA-expressing cells were generated. Similarly, MCL-1-silenced OCI-AML3 cells were generated by infecting a lentivirus carrying MCL-1-specific shRNA (nucleotides 2421-2440, RefSeq NM021960). As controls, each cell line was transduced with a lentivirus carrying shRNA targeting green fluorescent protein (GFP) in pLKO.1 (Addgene, Cambridge, MA). HL-60 cells with stable overexpression of either BCL-XL or BCL-2, or with an empty vector, were kindly provided by Dr. Kapil N. Bhalla (Baylor College of Medicine and Methodist Hospital Research Institute, Houston, TX).

**Antibodies for immunoblot analysis.**

Antibodies used for immunoblot analysis consisted of mouse monoclonal anti-p53 (DO-1; purchased from Santa Cruz Biotechnology, Santa Cruz, CA), mouse monoclonal anti-BCL-2 (124; purchased from Dako, Carpinteria, CA), rabbit monoclonal anti-BCL-XL (54H6; purchased from Cell Signaling Technology, Beverley, MD), mouse monoclonal anti-MCL-1 (22; purchased from BD Bioscience, San Jose, CA), and mouse monoclonal anti-β-actin (purchased from Sigma-Aldrich) antibodies.
